# Estimating V̇O_2_ max in healthy subjects without maximal effort: a novel protocol using ballistocardiography

**DOI:** 10.3389/fspor.2025.1717782

**Published:** 2026-01-15

**Authors:** Amin Hossein, Jérémy Rabineau, Philippe Questel, Adam Kobbai, Pierre-François Migeotte, Cyril Tordeur, Paniz Balali, Elza Abdessater, Alexis Gillet, Benoit Haut, Philippe van de Borne, Vitalie Faoro

**Affiliations:** 1 LPHYS, Department of Cardiology, Erasme University Hospital, Brussels, Belgium; 2 Research Unit in Cardio-respiratory Physiology, Exercise & Nutrition, Faculty of Human Movement Sciences, Université Libre de Bruxelles, Brussels, Belgium; 3 Department of Kinesiology and Health Sciences, University of Waterloo, Waterloo, ON, Canada; 4 TIPS, École Polytechnique de Bruxelles, Université Libre de Bruxelles, Brussels, Belgium

**Keywords:** seismocardiography, cardiopulmonary exercise testing, cardiorespiratory fitness, digital health, exercise physiology, non-invasive monitoring, stroke volume, submaximal exercise testing

## Abstract

Low maximal oxygen uptake (V̇O_2_ max) is a strong predictor of cardiovascular morbidity and mortality, yet its gold-standard assessment through cardiopulmonary exercise testing (CPET) requires maximal effort and specialized equipment. This study evaluates whether ballistocardiography (BCG), recorded during brief stabilization breaks embedded in a submaximal cycling protocol, can provide reliable estimates of V̇O_2_ max. BCG provides unique insights into stroke volume and blood displacement, offering a robust physiological basis for V̇O_2_ max estimation. Sixteen healthy young adults completed three randomized exercise protocols on a cycle ergometer with simultaneous gas-exchange analysis: a standard incremental step CPET until exhaustion (S) and two modified protocols including short breaks (B1 and B2) designed to facilitate high-quality BCG acquisition. BCG-derived kinetic output (KVO_2_) was used to predict V̇O_2_ max through a linear regression model based on early-stage workload increments. Both break-based protocols yielded CPET-measured V̇O_2_ max and maximal workload values comparable to those from the standard test. BCG-based V̇O_2_ max estimation, using only the first breaks of the protocol, which require a total exercise duration of 10.1 (9.5; 10.8) minutes without reaching maximal effort, demonstrated accuracy comparable to maximal standard tests, with a coefficient of variation of 12.05% and a mean absolute percentage error of 15.59%. While this study was limited to healthy young adults, the proposed approach holds potential for broader applications, particularly in diverse settings or in populations where maximal effort is impractical. Future work should focus on integrating additional BCG signal features and validating these methods in diverse populations.

## 1 Introduction

Cardiorespiratory fitness (CRF) is a crucial predictor of cardiovascular disease (CVD) risk (1). Recognizing this, the European Society of Cardiology (ESC), in its 2020 scientific position statement, advocated for integrating CRF evaluation into clinical settings to enhance CVD risk prediction and patient health management (2). The gold standard for CRF assessment is the direct measurement of maximal oxygen uptake (V̇O_2_ max) through breath-by-breath gas exchange during incremental exhaustive exercise (3). However, this approach can be impractical due to its demand for maximum physical effort from the participant, specialized equipment, trained personnel, and considerable time commitment. V̇O_2_ max can also be estimated through maximal or submaximal tests employing indirect methods with varying degrees of accuracy (4). In contrast, indirect methods, including field tests, estimate CRF using variables such as heart rate (HR), distance covered, or trial time. Field tests offer several advantages, including low cost, ease of implementation, accessibility, and the ability to evaluate large groups simultaneously. However, these tests rely on mathematical models and indirect estimations, which can introduce significant measurement errors (5). The importance of accurate estimation of CRF is underlined by the considerable improvement in survival (10%–25%) with only a small change in V̇O_2_ max of 1 metabolic equivalent (MET), corresponding to 3.5 mL·kg^−1^·min^−1^ (6).

Astrand's work in 1976 (7), in accordance with the Fick formula, highlighted how exercise-driven oxygen demand is satisfied through increased cardiac output and a widened arteriovenous oxygen difference. This relationship demonstrates that cardiac output increases proportionally to oxygen consumption (VO_2_) at exercise, with maximal VO_2_ also linearly correlated with maximal cardiac output. This interplay underscores the central role of the cardiovascular system in governing oxygen delivery to active tissues, establishing it as the primary determinant of aerobic capacity in healthy subjects, with mitochondrial utilization playing a secondary role in maximal aerobic exercise limitation (8). This foundational understanding of the cardiovascular system's role in oxygen consumption has led researchers to explore whether resting and submaximal cardiovascular parameters can reliably predict V̇O_2_ max (9–11).

Recent advancements in wearable sensor technology have introduced innovative approaches to assessing the cardiac status, particularly cardiac output, using ballistocardiography (BCG) (12, 13) and seismocardiography (SCG) (12, 14, 15). BCG and SCG capture subtle mechanical vibrations of the body generated by cardiac contractions and blood flow in central arteries. BCG measures these hemodynamic movements at the body's center of mass, while SCG records vibrations, closer to the heart, on the chest wall. From BCG, the time integral of kinetic energy (iK) over the cardiac cycle has been introduced as a measure of total cardiac mechanical effort. Metrics derived from iK were shown to be reproducible (16). In addition, differences in iK metrics have been associated with differences in stroke volume (SV), left ventricular ejection fraction, and cardiac output (CO) in healthy subjects (12, 17).

SCG has previously been used in studies involving exercise testing. Libonati and coworkers found that higher exercise capacity, defined as time to exhaustion on a treadmill test, correlated with a lower Tei Index derived from the SCG (18, 19). Recently, a resting SCG signal was used to build a model with other parameters, such as gender, age, and weight, to estimate the VO_2_ peak in healthy subjects (20). The reliability of this model estimation was shown to be high between test days. However, it underestimated the VO_2_ peak compared to the gold standard measurement (21, 22). On the other hand, studies have shown that some BCG features can be closer to SV than those derived from SCG (12). As SV max is an essential determinant of V̇O_2_ max, BCG shows potential for improving V̇O_2_ max prediction. Training-induced changes in V̇O_2_ max largely depend on adaptations in SV max rather than HR, further highlighting the importance of non-invasive SV assessment for monitoring cardiovascular fitness improvements (23). However, to our knowledge, no studies have been performed to estimate V̇O_2_ max based on these BCG features.

This study investigates the feasibility of estimating V̇O_2_ max using BCG during cardiopulmonary exercise testing (CPET) on a cycle-ergometer. To reduce motion artifacts and improve the quality of BCG signal acquisition, two alternative protocols were developed, each introducing short breaks after every incremental exercise step. These breaks allow participants to remain still, minimizing movement-related disturbances and enhancing the extraction of SV and CO features from the BCG signals. These two break-based protocols (B1 and B2) were compared to a conventional, continuous protocol without pauses (S), allowing us to assess the impact of step duration and recovery periods on both BCG signal accuracy and CPET-derived parameters.

To systematically evaluate the potential of this BCG-adapted approach for V̇O_2_ max estimation, a three-step methodology was followed. First, the consistency of CPET metrics obtained from B1 and B2 was assessed against those from the standard protocol S, with all participants performing the three protocols in randomized order. Second, the feasibility of predicting V̇O_2_ max from submaximal BCG-derived features was explored. Finally, traditional predictive models were re-implemented and benchmarked against the proposed BCG-based approach.

## 2 Methods

### 2.1 Participants

Sixteen healthy, active young adults (eight males and eight females) volunteered to participate in this study. None had specific pathologies or counter-indications preventing them from reaching V̇O_2_ max. The median (interquartile range) for age, body mass, body height, and body mass index (BMI) were 23.2 (22.9; 27.9) years, 64.0 (60.0; 70.0) kg, 172.5 (166.5; 177.5) cm, and 21.4 (20.6; 22.5) kg/m^2^, respectively (Table 1). The study complied with the Declaration of Helsinki and was approved by the local institutional review board (Hôpital Erasme—CCB: B4062020000539). Each subject provided written informed consent upon arrival for their first test. Within the scope of this pilot study, the sample size was not determined by an *a priori* power analysis but was estimated based on the number of participants typically included in similar pilot studies with comparable designs and objectives.

**Table 1:** Participants demographic.

Parameter	Population (*N* = 16)
Female, *n* (%)	8.0 (50.0)
Age, years [median (IQR)]	23.2 (22.9; 27.9)
Body mass, kg [median (IQR)]	64.0 (60.0; 70.0)
Body height, cm [median (IQR)]	172.5 (166.5; 177.5)
Body mass index, kg/m² [median (IQR)]	21.4 (20.6; 22.5)

IQR, interquartile range.

### 2.2 Experimental design

Three exercise protocols were designed to evaluate the feasibility of V̇O_2_ max estimation using BCG under different conditions. The gold-standard protocol, S, served as reference, with no breaks, following established guidelines for maximal exercise protocols to provide a baseline for comparison. B1 was designed to include the maximum number of breaks possible, allowing for the largest number of steps during which BCG measurements could be acquired while still aiming to achieve a reliable V̇O_2_ max measurement. Finally, B2 was developed as a trade-off, incorporating a minimal number of breaks (three to four), to balance the acquisition of BCG data with the likelihood of obtaining accurate V̇O_2_ max results in cases where B1 might not provide sufficient reliability.

Therefore, the participants visited the laboratory on three occasions. During each visit, they were equipped with the Kinocardiograph as previously described (24), measuring ECG at 200 Hz and linear accelerations and angular rates at 50 Hz. They were then asked to perform one of the three protocols (S, B1, or B2) in a randomized order using a cycle ergometer (Ergoselect II 1200; Ergoline, Bitz, Germany) until exhaustion.

Participants performed the tests in a standard upright seated position on a commercially available cycle ergometer, following conventional CPET posture and setup. BCG was recorded using a single inertial measurement unit positioned over the lower back at the level of the lumbar spine (L3–L5 region), a common location for capturing whole-body cardiomechanical motion. The sensor was secured with a strap. No additional harnesses or non-standard equipment were required.

For each subject, these protocols were all completed within three weeks to avoid potential changes in overall cardiorespiratory fitness. The protocols were separated by at least 48 h to ensure adequate physiological recovery, and they were performed at the same time of the day for a given participant to minimize circadian variance. In addition, the participants were asked to refrain from eating and consuming caffeine during the two hours before each protocol and to avoid intense physical activity during the 24 h before each protocol. The data were collected at the Erasme Hospital (Brussels, Belgium).

**Figure 1:**
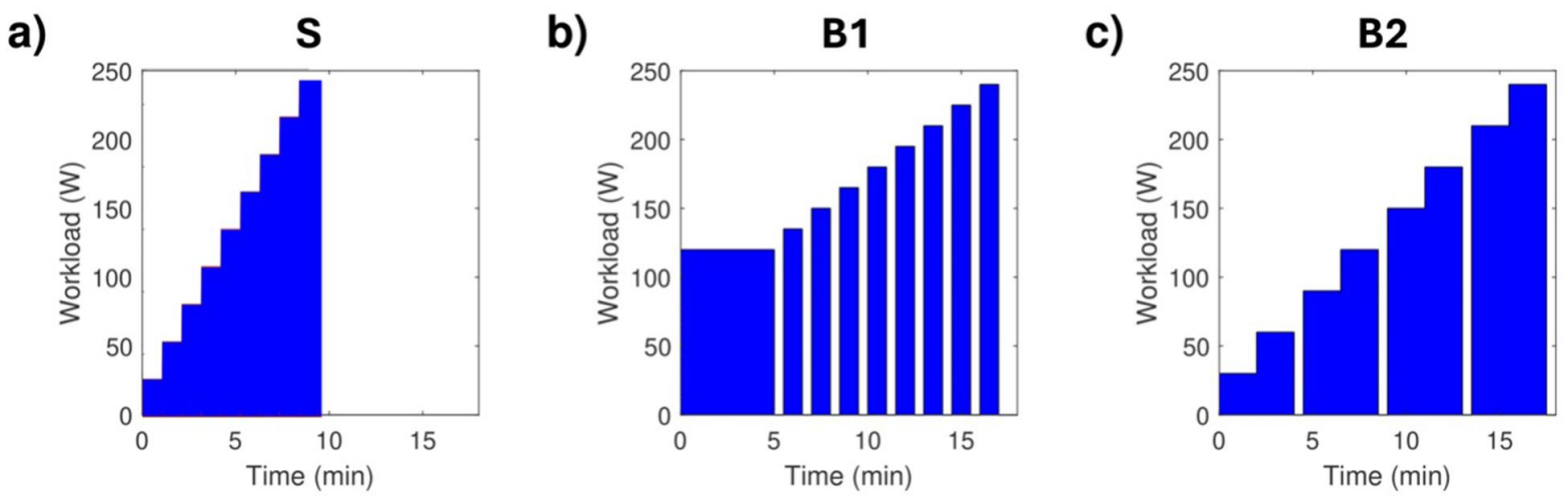
Graphical representation of the evolution of workload for the three tested protocols in the case of a male subject reaching 240 W: **(a)** Protocol S; **(b)** Protocol B1; **(c)** Protocol B2.

Each protocol started with a 1 min record at rest while the subject sat on the cycle ergometer. The S protocol consisted of a standard incremental step exercise test (4). Both female and male subjects started at a minimal workload (20 W and 30 W, respectively) with no warm-up period. Afterwards, the subjects observed increments in workload every 60 s until exhaustion (20 W or 30 W for female or male subjects, respectively; see example in Figure 1a) (4). The protocols with breaks were designed to fulfil the dual aim of this study: enabling high-fidelity BCG signal acquisition during dynamic exercise and allowing accurate comparison with V̇O_2_ max. Protocols B1 and B2 introduced regular 30 s breaks to create periods of reduced motion, improving thoracic stability and minimizing artifacts critical for reliable BCG measurements. Physiologically, both protocols maintain progressive cardiovascular loading. B1 includes frequent breaks between each workload increment, facilitating the isolation of responses at specific intensities. In contrast, B2 features fewer breaks with longer workload durations, promoting metabolic stabilization at each step and supporting accurate gas exchange analysis. The B1 protocol started with a 5 min warm-up (at 80 W or 120 W for female or male subjects, respectively), with increments in workload every 60 s until exhaustion (10 W or 15 W for female or male subjects, respectively, see example in Figure 1b). In addition, a 30 s break was observed between each increment. Finally, for the B2 protocol, female and male subjects started at a minimal workload (20 W and 30 W, respectively) with no warm-up period. Then, the subjects observed increments in workload every 120 s until exhaustion (20 W or 30 W for female or male subjects, respectively; see example in Figure 1c). In addition, a 30 s break was observed every second increment.

The participants were asked to keep a cadence of 70–90 rpm during each protocol, except during the breaks, when no movements were allowed. ECG and arterial oxygen saturation (SpO_2_) were continuously monitored, and blood pressure was measured every 2 min by sphygmomanometer. Data were collected through a tightly fitted facial mask and analysed breath by breath using a metabolic system (Exp'Air, Medisoft, Dinant, Belgium) calibrated with room air and standardized calibration gas following the manufacturer's recommendations. V̇O_2_ max was considered to be achieved when three of the following criteria were met: an increase in VO_2_ of less than 100 mL/min with a further increase in workload (or, in protocols including breaks, no further VO_2_ increase after starting a new increment), respiratory exchange ratio (RER) greater than 1.15, achievement of age-predicted maximal heart rate, and the incapacity of the subject to maintain the required pedalling frequency despite verbal encouragement. Maximum workload (W_max_) was defined as the highest workload sustained (in watts) and measured as the last workload increment the subject completed successfully. Maximal heart rate (HR_max_) was defined as the highest HR recorded during the test. Dedicated software (Exp'Air, Medisoft, Dinant, Belgium) was used to display data numerically and graphically in real time. After each incremental step, the participants were asked to rate their subjective level of exertion on a scale from 6 to 20, based on the Borg Rating of Perceived Exertion (RPE) scale (25, 26).

### 2.3 Data processing

As described in previous publications (16, 24), the linear kinetic energy (K) signal was computed using the Newtonian equations. To achieve this, the mass of the Kinocardiograph was used as the inertial parameter, Equation (1).

$$K=\frac{1}{2}m(v_{x}^{2}+\;v_{y}^{2}+\;v_{z}^{2})$$ (1)

where *K* is the linear kinetic energy, *m* is the mass of the sensor; $v_{x}$, $v_{y}$, and $v_{z}$ are the orthogonal components of the velocity vector $\vec{v}$.

The linear kinetic energy was processed using a centred moving average with a window length of 5 s and a step size of 1 s, providing the average energy (in joules) within each 5 s interval. This signal was then multiplied by 60 to obtain a “kinetic output” $\left(K_{out}\right)$ (J/min), Equation (2).

$$K_{out}(t)=\int_{t-2.5}^{t+2.5}\frac{K(t).dt}{5}.60\;$$ (2)

For each period of measurement, corresponding to the stops of the protocols B1 and B2, the median of $K_{out}$ was stored, together with the corresponding median heart rate (HR, in beat/min). No automated beat-level quality control or rejection criteria were applied. All beats within each 30 s break were included, and the median $K_{out}$ value was used to mitigate the influence of occasional noisy beats. Visual inspection confirmed that more than 95% of beats were stable and free of major artifacts during breaks.

The average kinetic energy generated per heartbeat ($K_{b}$, in J/beat) is proportional to the ejected blood mass and the square of velocity. Blood velocity is, according to the Frank-Starling's law (27), in turn, proportional to the blood volume within the left ventricle before ejection, which directly correlates with stroke volume. Therefore, greater ventricular filling leads to a higher stroke volume and increased kinetic energy per beat.

$$K_{b}\propto mv^{2}\propto m^{3}\propto SV^{3}$$ (3)

As $K_{out}\propto K_{b}HR$, we have $SV\propto \sqrt[{3}]{K_{out}/HR}$. Thus, when this stroke volume-derived metric is subsequently multiplied by the heart rate, it becomes proportional to CO, which is proportional to the oxygen uptake

$$K_{VO2}=\sqrt[{3}]{\frac{K_{out}}{HR}}\cdot HR\;\propto CO\;\propto \;VO_{2}$$ (4)

**Figure 2:**
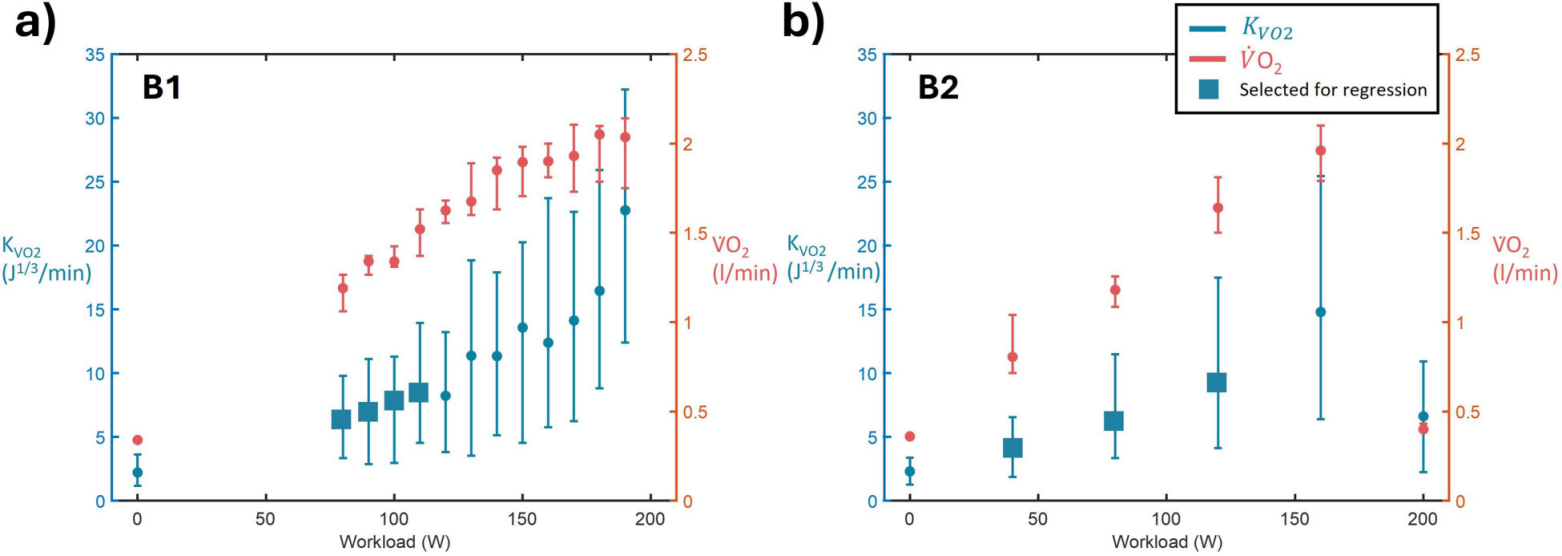
Graphical representation, from one representative subject, of the steps selected for the linear regression for the two protocols with breaks: **(a)** Protocol B1; **(b)** Protocol B2. Medians and interquartile ranges for $K_{VO2}$ are represented in blue, while they are represented in red for VO_2_. The blue square fill represents the steps selected to compute the linear regression as described in Annex A.

To estimate V̇O_2_ max based on $K_{VO2}$, a linear regression model between $K_{VO2}$ and the workload at each step of the protocols B1 and B2 was built. This linear model is based only on the first workloads, as the relationship is non-linear after a certain workload, specific to each participant. As there are only 3–5 steps for the B2 protocol, the first three are considered to build the linear regression. For B1, participants performed between 12 and 16 steps. In that case, we identified the linear region for regression modelling by dynamically evaluating the relationship between kinetic output and the workload while accounting for potential deviations from linearity. Successive steps were added until linearity criteria were no longer met. Linearity was defined as a coefficient of determination R² ≥ 0.85 and a maximum pointwise residual <15%. In protocol B1, this typically corresponded to the first 3–7 breaks. This method is further exposed in Annex A. Examples of linear segments during protocols B1 and B2 for a single subject are presented in Figure 2.

The estimation of V̇O_2_ max leverages the linear relationship between $K_{VO2}$ and external workload in the moderate-intensity domain. To establish this relationship, a median ratio R_S_ was calculated from the zone of linear evolution of VO_2_ and K_VO2_:

$$R_{S}=median\left(\frac{\dot{V}O_{2}}{K_{VO2}}\right)$$ (5)

This ratio serves as a scaling factor. The linear regression model was applied to the K_VO2_ data points, enabling the prediction of $K_{VO2}$ at maximal workload (W_max_):

$$(K_{VO2}{)}_{max}=a.W_{max}+b$$ (6)

where *a* and *b* represent the slope and intercept of the regression line, respectively. Using this model, the predicted maximal $K_{VO2}$ value was scaled by $R_{S}$ to provide an initial estimate of V̇O_2_ max:

$$(\dot{V}O2_{max}{)}^{est}=\;(K_{VO2}{)}_{max}\cdot R_{S}$$ (7)

Equation (7) provides the foundation for evaluating V̇O_2_ max, assuming all parameters in the equation are known. However, the objective is to assess cardiorespiratory fitness without needing a full maximal effort test, meaning that the maximal workload is not directly measured. Instead, the maximum workload can be estimated based on the subject's measured maximal heart rate during the corresponding test. This maximal heart rate can subsequently be estimated using established formulas, thereby enabling the estimation of maximal workload without the need for direct measurement. The results derived from Equation (7) are presented under three conditions: (1) using the actual measured maximal workload (W_max_); (2) using W_max_ estimated from the measured maximal heart rate (HR_max_); and (3) using W_max_ estimated from an age-predicted HR_max_ (HR_est_ = 220−age).

### 2.4 Other VO_2_ prediction models

In the past, different methods have been developed to estimate V̇O_2_ max when gas analysis is unavailable. They can be separated into three categories: maximal exercise testing, submaximal exercise testing, and non-exercise prediction (6). The non-exercise prediction equations from the FRIEND study (28) and WASSERMAN (4), as well as maximal exercise-based models including FRIEND-ergometry (29), the Military Fitness Test (MILFIT) (30), and the STORER studies (31), were applied to the raw data of participants in this study to compare their predictive performance with the model proposed in this work. The equations are described in Annex B.

### 2.5 Statistical analysis

All the statistical analyses were performed using GraphPad Prism 9.5.1 (GraphPad Holdings, LLC, Boston, MA, USA) and Matlab R2023a (The MathWorks, Inc., Natick, MA, USA). Statistical significance was considered for *p*-values below 0.05.

The B1 and B2 protocols were compared to the reference S for each feature of interest (total duration, W_max_, V̇O_2_ max, and HR_max_). To compare the mean ranks, a Friedman test was used, together with a two-stage step-up method of Benjamini, Krieger, and Yekuteli to account for multiple comparisons. A Bland-Altman analysis was conducted to extract the bias and 95% limits of agreement after ensuring that the differences followed a normal distribution, using an Anderson–Darling test. V̇O_2_ max and Wmax differences required log-transformation prior to correlation analyses. The strength of the relationship was evaluated by computing Pearson's correlation coefficient r and the associated *p*-value after ensuring that the samples followed a bivariate normal distribution using a Royston test. When this was not the case, these parameters were evaluated after the log transformation of the data. The general agreement was evaluated by computing the coefficient of variation (CV), using a root mean square approach and the MAPE. Finally, Lin's concordance correlation coefficient (CCC) and 95% confidence interval were also computed. Since CCC is very similar to the intraclass correlation coefficient, another metric of agreement between two measurements, it is thus possible to apply the same guidelines for their interpretation: values less than 0.5, between 0.5 and 0.75, between 0.75 and 0.9, and greater than 0.90 are indicative of poor, moderate, good, and excellent reliability, respectively. For all experiments, all outcome measures, experimental conditions, and data exclusions were fully reported, with no unreported analyses or selective omission of results.

## 3 Results

### 3.1 Impact of breaks on exercise performance

**Figure 3:**
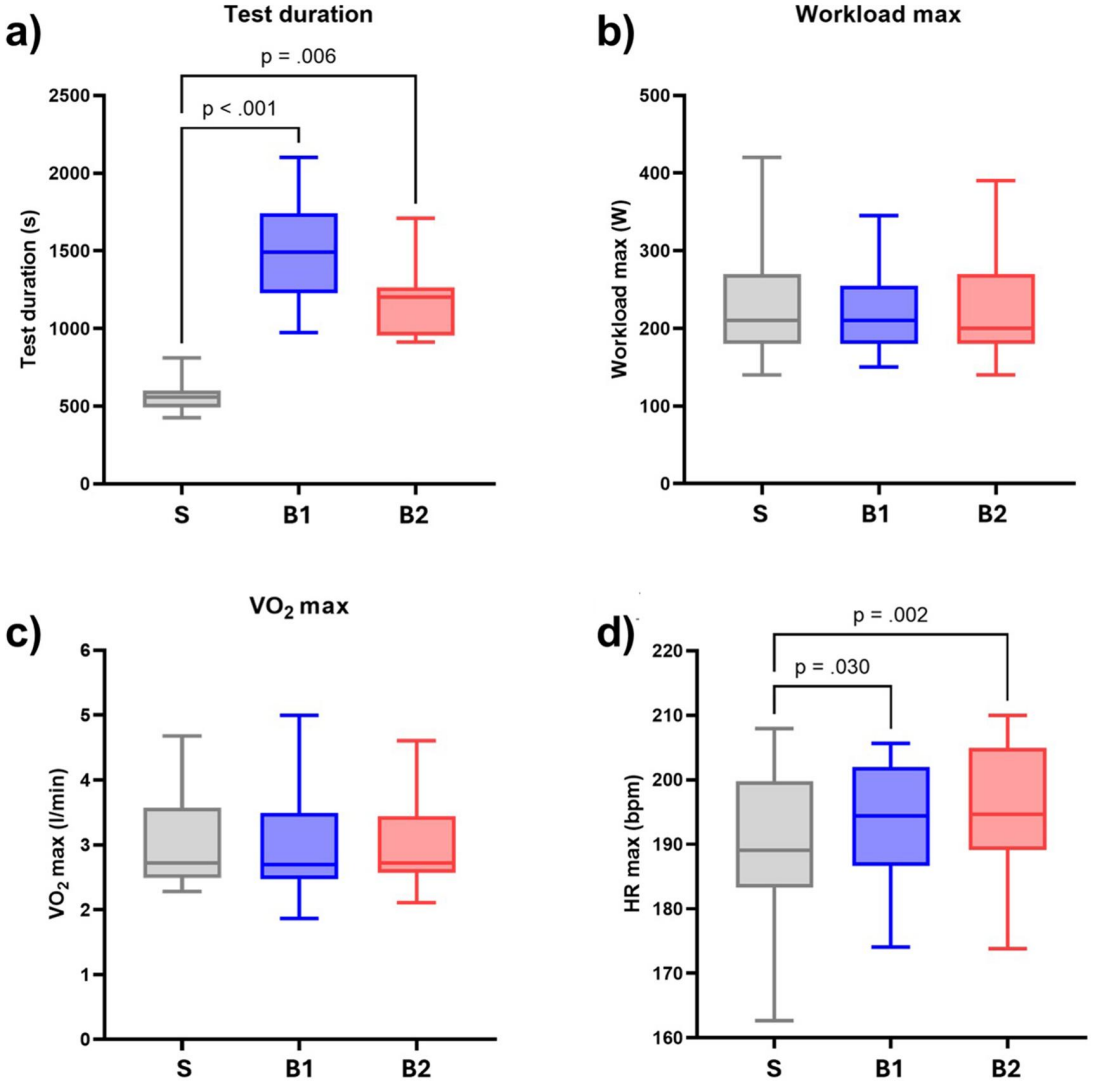
Box-and-whisker plots representing the full cohort (*n* = 16), comparing the protocols S, B1, and B2 for: **(a)** test duration; **(b)** maximum workload; **(c)** maximum oxygen uptake; and **(d)** maximum heart rate. The *p*-values correspond to the comparison of the median between the protocols, using a Friedman test and the two-stage step-up method of Benjamini, Krieger, and Yekuteli to account for multiple comparisons. V̇O_2_ max, maximal oxygen uptake; HR, heart rate. All values correspond to direct CPET measurements.

One participant had to be excluded from the inter-protocol comparison because 27 days separated the first and last records, exceeding the 21-day threshold. However, this participant was retained for all subsequent analyses. Across the entire cohort, the median (IQR) time between the first and last record was 15 (12; 19) days. In order to compare the effect of the different protocols, the results of the direct CPET measurements related to test duration, W_max_, V̇O_2_ max, and HR_max_ for the three protocols are given in Figure 3. The test duration was almost three times longer for B1 (*p* < 0.001), and almost twice longer for B2 (*p* = 0.006), than for S. HR_max_ was also slightly higher for B1 (*p* = 0.030) and for B2 (*p* = 0.002) than for the reference protocol (see the corresponding biases in Tables 2, 3). However, no statistical differences were observed between any protocols for W_max_ and VO_2_ max.

**Table 2:** Comparison of the different features of interest for protocol B1 vs. protocol S.

B1 vs. S (*n* = 15)	Bias (95% limits of agreement)	Pearson's *r* and *p*	Lin's CCC (95% confidence interval)	Coefficient of variation (%)	MAPE (%)	SEE
Test duration (s)	+939 (+467; +1,412)*	*r* = 0.86; *p* < 0.001	0.05 (0.01; 0.09)	63.9	61.9	1,039.8
Workload max (W)	−10 (−58; +39)^†^	*r* = 0.97; *p* < 0.001	0.92 (0.84; 0.96)	6.1	6.8	27.8
V̇O_2_ max (L/min)	−0.10 (−0.91; +0.72)^†^	*r* = 0.87; *p* < 0.001^‡^	0.86 (0.64; 0.95)	8.6	8.3	0.4
HR max (bpm)	+4 (−8; +15)*	*r* = 0.92; *p* < 0.001	0.84 (0.65; 0.93)	2.6	3.0	7.3

V̇O_2_ max, maximal oxygen uptake; HR, heart rate; CCC, concordance correlation coefficient; MAPE, mean absolute percent error; SEE, standard error of the estimate. Guidelines for interpretation of CCC: less than 0.5 (poor); from 0.5 to 0.75 (moderate); from 0.75 to 0.90 (good); more than 0.90 (excellent). The coefficient of variation (CV) is computed using the root mean square approach.

* *p* < 0.05 when comparing the median to the one of S, using a Friedman test and the two-stage step-up method of Benjamini, Krieger, and Yekuteli to account for multiple comparisons.

† Limits of agreement are given indicatively, as the differences do not follow a normal distribution.

‡ Computed after log transformation.

**Table 3:** Comparison of the different features of interest for protocol B2 vs. protocol S.

B2 vs. S (*n* = 15)	Bias (95% limits of agreement)	Pearson's *r* and *p*	Lin's CCC (95% confidence interval)	CV (%)	MAPE (%)	SEE
Test duration (s)	+598 (+333; +862)*	*r* = 0.90; *p* < 0.001	0.09 (0.02; 0.15)	49.0	51.1	657.1
Workload max (W)	−7 (−34; +21)^†^	*r* = 0.98; *p* < 0.001	0.97 (0.94; 0.99)	4.9	4.5	16.2
V̇O_2_ max (L/min)	−0.05 (−0.86; +0.78)	*r* = 0.85; *p* < 0.001	0.85 (0.62; 0.95)	8.7	10.5	0.4
HR max (bpm)	+5 (−6; +17)*	*r* = 0.91; *p* < 0.001	0.81 (0.59; 0.92)	2.9	3.2	8.2

V̇O_2_ max, maximal oxygen uptake; HR, heart rate; CCC, concordance correlation coefficient; MAPE, mean absolute percent error; SEE, standard error of the estimate. Guidelines for interpretation of CCC: less than 0.5 (poor); from 0.5 to 0.75 (moderate); from 0.75 to 0.90 (good); more than 0.90 (excellent). Coefficient of variation (CV) computed using the root mean square approach.

* *p* < 0.05 when comparing the median to the one of S, using a Friedman test and the two-stage step-up method of Benjamini, Krieger, and Yekuteli to account for multiple comparisons.

† Limits of agreement given indicatively, as the differences do not follow a normal distribution.

Table 2 reports features of interest to assess the overall agreement between the protocols B1 and S. Table 3 reports the same features for protocol B2 vs. S. A strong correlation (Pearson's *r* > 0.85, *p* < 0.001) was found for all the parameters, both when testing B1 vs. S and B2 vs. S. Lin's CCC for test duration (0.05 and 0.09 for B1 and B2, respectively), confirms that, even though strongly correlated, the test duration was different between the protocols. However, for the other parameters, the concordance given by Lin's CCC was found to be good for V̇O_2_ max (0.86 and 0.85 for B1 and B2, respectively) and HR_max_ (0.84 and 0.81 for B1 and B2, respectively), and even excellent for W_max_ (0.92 and 0.97 for B1 and B2, respectively). The same results were also confirmed when looking at the coefficients of variation (see Tables 2, 3 for B1 and B2, respectively), indicating large differences for the test duration but good agreement for V̇O_2_ max, HR_max_, and W_max_. At maximum effort, The Borg RPE scores for the protocols were 19.5 (18.5, 20.0) for S, 20.0 (19.5, 20.0) for B1, and 20.0 (19.5, 20.0) for B2. Notably, S demonstrated significantly lower perceived exertion compared to both B1 (*p* < 0.01) and B2 (*p* < 0.001).

### 3.2 Comparison of V̇O_2_ max estimation methods

Table 4 compares various V̇O_2_ max estimation methods. Among the models that do not require W_max_ to be measured, the FRIENDS model exhibits the lowest bias of 0.03 [95% limits of agreement: (−0.96; 1.02)] and achieves a strong Pearson's correlation (*r* = 0.77, *p* < 0.001) with a Lin's CCC of 0.75 [95% CI: (0.43; 0.90)]. It also demonstrates a moderate CV of 11.11% and an MAPE of 12.60%. By comparison, WASSERMAN has a slightly higher bias [−0.57 (−1.47; 0.34)] but shows a stronger correlation (*r* = 0.82, *p* < 0.001). However, its lower CCC [0.57 (0.27; 0.77)] and higher CV (18.00%) and MAPE (26.05%) suggest reduced precision compared to FRIENDS.

**Table 4:** Comparison of V̇O_2_ max estimation methods in the present data set: bias, limits of agreement, Pearson's correlation coefficient (*r*) and associated *p*-values, Lin's concordance correlation coefficient (CCC) with 95% confidence intervals, coefficient of variation (CV), and mean absolute percentage error (MAPE), standard error of the estimate (SEE).

V̇O_2_ max estimation	Input Parameters	Bias (95% limits of agreement)	Pearson's *r* and *p*	Lin's CCC (95% confidence interval)	CV (%)	MAPE (%)	SEE (L/min)
FRIEND	Anthropometrics	−0.03 (−1.04; 0.99)	*r* = 0.74; *p* < 0.001	0.72 (0.37; 0.89)	11.64	13.06	0.54
WASSERMAN	Anthropometrics	−0.57 (−1.47; 0.34)	*r* = 0.82; *p* < 0.001	0.57 (0.27; 0.77)	18.00	26.05	0.77
FRIEND-ERGO	W_max_	−0.39 (−1.04; 0.27)	*r* = 0.91; *p* < 0.001	0.78 (0.54; 0.90)	12.18	15.56	0.54
MILFIT	W_max_	0.03 (−0.61; 0.67)	*r* = 0.91; *p* < 0.001	0.90 (0.75; 0.96)	7.24	9.32	0.34
STORER	W_max_	−0.14 (−0.83; 0.55)	*r* = 0.90; *p* < 0.001	0.89 (0.70; 0.96)	8.96	10.86	0.39
BCG with protocol B1	W_max_	−0.16 (−0.93; 0.61)	*r* = 0.93; *p* < 0.001	0.89 (0.74; 0.96)	9.54	13.12	0.44
	HR_max_	−0.17 (−0.94; 0.60)	*r* = 0.94; *p* < 0.001	0.89 (0.75; 0.95)	9.82	13.24	0.45
	HR_est_	−0.13 (−1.06; 0.80)	*r* = 0.88; *p* < 0.001	0.85 (0.63; 0.94)	12.05	15.59	0.51
BCG with protocol B2	W_max_	−0.22 (−1.92; 1.48)	*r* = 0.58; *p* < 0.001	0.52 (0.10; 0.79)	19.16	24.09	0.93
	HR_max_	−0.36 (−1.69; 0.97)	*r* = 0.62; *p* < 0.001	0.53 (0.12; 0.79)	16.90	23.08	0.80
	HR_est_	−0.32 (−1.91; 1.26)	*r* = 0.47; *p* < 0.001	0.43 (−0.04; 0.75)	18.41	24.57	0.91

The methods include standard predictive models (FRIENDS, WASSERMAN, FRIEND-ERGO, MILFIT, and STORER) and ballistocardiography (BCG)-based estimates using Protocols B1 and B2 under different inputs: maximal workload (W_max_), measured maximal heart rate (HR_max_), and estimated maximal heart rate (HR_est_). Lower bias, narrower limits of agreement, and lower CV, SEE and MAPE values indicate higher precision and accuracy.

For methods requiring W_max_ as an input, MILFIT achieves one of the best results with a bias of 0.03 (−0.61; 0.67), an excellent Pearson's correlation (*r* = 0.91, *p* < 0.001), and a CCC of 0.90 (0.75; 0.96). Its low CV (7.24%) and MAPE (9.32%) further underscore its accuracy. FRIEND-ERGO and STORER also perform well, with biases of −0.39 (−1.04; 0.27) and −0.14 (−0.83; 0.55), respectively, and comparable CCC values around 0.89.

### 3.3 V̇O_2_ max estimations with ballistocardiography

As described in the methods, 3 steps were used to build the linear regression for protocol B2, accounting for 13.2 (11.2; 14.3) minutes to estimate V̇O_2_ max with BCG. On the other hand, the method used to extract the linear regression from the steps within protocol B1 isolated between 3 and 7 steps, accounting for a mean duration of 10.1 (9.5; 10.8) minutes.

Using Protocol B1, the BCG methods achieved strong performance, particularly when HR_max_ or W_max_ were used as inputs. The mean biases range from −0.17 to −0.13. Pearson's correlation coefficient (*r*) was consistently high (0.88–0.94, *p* < 0.001), and Lin's CCC remained strong, ranging from 0.85 to 0.89, thus indicating strong agreement between predicted and actual values.

In contrast, the B2 BCG protocols underperformed relative to B1. The biases were higher, ranging from −0.36 to −0.22, with broader limits of agreement and weaker correlation coefficients (*r* = 0.47–0.62, *p* < 0.001). Lin's CCC was notably lower, with the highest value for B2 being 0.53 (0.12; 0.79) when using HR_max_. The CV and MAPE values were also substantially higher for B2, indicating reduced agreement. The results for an estimation of V̇O_2_ max normalized by body mass are presented in Supplementary Appendix C, Supplementary Table C1, displaying similar findings.

## 4 Discussion

This randomized study introduces a novel exercise test protocol incorporating breaks, enabling accurate V̇O_2_ max estimation while facilitating simultaneous BCG measurements during these breaks. Our findings demonstrate that BCG-derived V̇O_2_ max estimation using an age-based approximation of maximal heart rate yields reliable results, particularly when limited to the initial breaks of the B1 protocol. Notably, we found that a submaximal test lasting approximately 10 min was sufficient to achieve accurate BCG-derived V̇O_2_ max estimates. These results underscore the potential of wearable BCG technologies to streamline cardiorespiratory fitness assessment by overcoming the logistical and physical challenges of traditional maximal effort testing while maintaining a high level of measurement accuracy.

### 4.1 Comparison of protocols to estimate exercise parameters

When comparing the standard protocol without breaks (S) to those incorporating breaks (B1 and B2), no significant differences were observed in V̇O_2_ max or W_max_. Both protocols demonstrated similar V̇O_2_ max estimation accuracy compared to S, with intraclass classifiers (ICC) of 0.86 for B1 and 0.85 for B2, and CVs of 8.6% and 8.7%, respectively. These findings are consistent with the literature, including a study by Katch et al., in which participants completed an average of 16 maximal exercise tests over a 2–4-week period, yielding a total error of ±5.6%, with biological variability accounting for ∼93% and measurement error for ∼7% (32). Other studies have reported comparable CV values when comparing different protocols on cycle ergometers (31, 33). Given that the mean intervals between tests S and B1, and S and B2 were 8 and 7 days, respectively, the results align with the standard repeatability expected for such tests, suggesting that introducing breaks does not compromise the reliability of cardiorespiratory fitness or exertion capacity assessments in healthy subjects. This aligns with numerous early studies comparing discontinuous and continuous protocols, reinforcing that intermittent breaks do not significantly impact the accuracy or reliability of V̇O_2_ max assessments (34–36).

Notably, B1 and B2 were associated with slightly larger values of HR_max_, approximately 4–5 bpm higher than S. This finding suggests that HR_max_ observed in S may not have been truly maximal, as it could still be improved in B1 and B2, just as a higher HR_max_ is typically observed in treadmill tests compared to cycle ergometer tests. Accounting for the lack of differences in V̇O_2_ max and W_max_ between protocols, several potential mechanisms may explain this observation. One possibility is that transient HR recovery during the break may have influenced baroreflex resetting or autonomic modulation, leading to an overshoot in sympathetic reactivation at the onset of the new workload, which would induce a higher HR response. Alternatively, a delayed restoration of venous return and SV after the break may have required a greater reliance on HR to maintain CO upon resumption of exercise.

At the peripheral level, slow O_2_ extraction kinetics after the break may have led to a transient compensatory increase in CO, primarily driven by HR, to sustain VO_2_ demand during the early phase of each step.

Finally, the additional recovery periods likely reduced fatigue and leg discomfort, enabling participants to sustain higher peak heart rates. However, these hypotheses warrant further investigation to elucidate the mechanisms underlying these changes. It should be noted that BCG-derived VO_2_ estimations were computed exclusively from the break periods, minimizing the influence of the changes in maximum HR between protocols. However, the latter may influence HR_max_-based workload estimations.

As expected, the inclusion of breaks extended the total protocol duration. While longer durations might be seen as a drawback, they provide the advantage of facilitating data collection during the breaks, particularly for methods like BCG that require the subject to remain still. Notably, for V̇O_2_ max estimation in B1, only the first 3–7 breaks were necessary, corresponding to a mean test duration of 10.1 min. By comparison, the mean duration of S was 9.2 min, suggesting that a submaximal protocol using only the initial breaks could achieve similar reliability with minimal additional time compared to a standard protocol. Moreover, the overall testing workflow may be further streamlined, as BCG-based protocols require less extensive instrumentation than standard CPET setups, potentially reducing the total preparation time and improving feasibility in practical settings.

### 4.2 Estimation of V̇O_2_ max

Submaximal exercise tests have been extensively studied due to their practicality and reduced physical demand compared to maximal testing. Submaximal exercise tests lasting under 5 min, such as the Single-Stage Submaximal Treadmill Test, demonstrate a mean CV between 10% and 12%, with standard errors of the estimate (SEE) typically around 4–7 mL/kg/min (37, 38). For protocols lasting less than 10 min, including the YMCA Cycle Test and the 1-Mile Jog Test, CV ranges narrow slightly (8%–10%), and SEE improves to approximately 4 mL/kg/min (33, 39). By way of comparison, maximal field tests such as the 12 min Run Test provide even greater accuracy, with CVs as low as 6%–8% and SEE below 3 mL/kg/min (30). For example, the Military Fitness test, which incorporates performance metrics from standardized physical activities, reported a correlation r between 0.80 and 0.84 with direct V̇O_2_ max in young recruits, with an ICC varying between 0.82 and 0.93 (30); but systematic biases rendered it less precise in older or less-trained populations (40). Non-exercise predictive equations, such as Wasserman's Equation, the FRIEND's Equation, and the Jackson Non-Exercise Test, rely on demographic and biometric data. While these methods offer low operational complexity and broader applicability, they present larger variability, with CV exceeding 15% and SEE averaging around 5–10 mL/kg/min, indicating lower reliability than performance-based tests (28, 41–43). While these field-based methods are practical, their accuracy is often population-specific, requiring calibration for target groups (44, 45).

BCG provides unique insights into blood displacement within the cardiac chambers and the large arteries of the body (13, 46), offering a robust physiological foundation for estimating V̇O_2_ max. Unlike traditional field methods that rely on external performance metrics or demographic data, BCG captures mechanical signals generated by cardiac contractions and blood flow, directly reflecting cardiovascular function. This intrinsic link to stroke volume makes BCG particularly well-suited for cardiorespiratory fitness assessments.

The results obtained in our study using BCG with protocol B1 demonstrate competitive accuracy when compared to submaximal exercise and field-based V̇O_2_ max estimation methods reported in the literature. Indeed, the BCG-based estimation with HR_est_ achieved a CV of 12.1%, a MAPE of 15.6%, and an SEE of 8.4 mL/kg/min (Table 4 and Supplementary Table C1), placing it within the range of values reported for submaximal tests lasting under 10 min, such as the YMCA Cycle Test and the 1-Mile Jog Test, which exhibit CVs between 9% and 12% and SEE around 4 mL/kg/min (47). While the B1 BCG models using HR_max_ or W_max_ yielded slightly higher accuracy, the HR_est_ approach eliminates the need for maximal effort testing, making it a practical alternative.

The accuracy of protocol B2 was lower (CV = 18.4%, MAPE = 24.6%), but these results remain comparable to those of anthropometrics-based methods like Wasserman's Equation re-implemented in this study (CV = 18.0%, MAPE = 26.1%). The lower accuracy of V̇O_2_ max estimations obtained from BCG metrics in the B2 protocol compared to B1 can be attributed to two potential factors. First, the greater spacing between breaks in B2 increases the likelihood that a break occurs beyond the first respiratory threshold. This transition marks a shift in metabolic dynamics, breaking the linear relationship between $K_{VO2}$ and VO_2_, which introduces bias into the regression model used for estimation. In contrast, B1 includes more frequent breaks, ensuring that the majority of data points used for regression remain within the linear phase of the $K_{VO2}/\dot{V}O_{2}$ relationship. Second, the limited number of data points in B2 (only three) makes the regression more sensitive to noise, as any deviation due to physiological variability, measurement error, or transient artifacts has a greater impact on the final estimation. In B1, where up to seven data points are available, the redundancy helps maintain robustness by mitigating the inﬂuence of individual outliers.

When re-implementing established predictive equations, such as FRIEND, MILFIT, and STORER, on our dataset, our findings aligned closely with previously reported performance metrics. For instance, the MILFIT equation achieved excellent reliability with a CV of 7.2% and MAPE of 9.3%, consistent with its original validation (30). Similarly, the FRIEND equation demonstrated moderate accuracy, with a CV of 11.6%, an MAPE of 13.1%, and a CCC of 0.72, outperforming BCG under protocol B2 but worse than BCG under protocol B1. However, the primary advantage of BCG-based methods lies in their reliance on performance measurements rather than purely demographic or biometric data. While the approach in this work was tested on healthy young individuals, it likely scales better to less conventional cases, such as populations with varying health conditions or fitness levels, compared to traditional equations designed and validated primarily for healthy young individuals. The BCG-based estimation, particularly under protocol B1, balances accuracy and feasibility. While not universally superior to all established methods, its adaptability, reliance on submaximal effort, and compatibility with wearable technology position it as a promising alternative for V̇O_2_ max estimation in diverse contexts, particularly when traditional testing methods are impractical.

### 4.3 Limitations and perspectives

This study includes several limitations that should be addressed in future work. First, while participants were instructed to remain still during breaks to facilitate accurate BCG measurements, maintaining complete stillness during exercise tests can be challenging. Nevertheless, as the analysis relies primarily on the initial steps of the protocol for regression modelling, the impact of this limitation may be mitigated. Future versions of this method should incorporate automated BCG quality indices to enable reproducible beat-level quality screening.

Second, the study was conducted on a small cohort of healthy young and active adults, limiting its generalizability. Further research with larger, more diverse populations, including children, the elderly, and patients with varying fitness levels or specific conditions, is essential. Indeed, sedentary individuals may experience greater dyspnea and larger chest movements during breaks, rendering acquisition less reliable. The submaximal nature of the proposed protocols offers significant potential for use in patient populations where maximal exertion tests are impractical. Additionally, even if the method does not provide an exact measurement of V̇O_2_ max, it holds significant potential for longitudinal use, whether to monitor training progress in athletes or to track recovery and rehabilitation in patients undergoing cardiac reconditioning.

Third, the estimation of V̇O_2_ max in this study involved a proportionality coefficient between VO_2_ and K_VO2_ assumed to be constant for each subject. While this parameter is likely linked to intrinsic physiological characteristics, it may differ in populations with altered ventricular compliance, chronotropic impairment, autonomic dysfunction, or earlier onset of anaerobic metabolism. Such factors may also reduce the linear zone or shift it to lower workloads. A formal sensitivity analysis of the linear-zone selection was not performed. The protocol provides only a few early workload steps (3–7 in B1), and the small cohort (*n* = 16) would make stepwise inclusion or exclusion largely driven by sampling noise rather than meaningful methodological variation. Larger studies covering a broader range of submaximal workloads will enable a more robust evaluation of linear-zone stability. These aspects warrant dedicated validation studies.

In this study, the integration of cardiac and central hemodynamic kinetic energy derived from the BCG signal demonstrated the feasibility of estimating V̇O_2_ max with reasonable accuracy. However, the potential of BCG extends beyond this initial application. By incorporating additional features of the BCG signal, such as specific waveform characteristics, amplitude variations, and timing parameters, future models could capture more nuanced aspects of cardiac dynamics at rest and exercise. These enhanced models may significantly improve the precision of V̇O_2_ max estimations, reducing variability and broadening applicability across different populations and conditions. An additional advantage of using BCG is its potential to provide detailed insights into the individual contributions of SV and HR to CO throughout the effort. This capability could enhance the understanding of cardiovascular dynamics during exercise and offer valuable physiological markers for fitness assessment and clinical evaluations.

Furthermore, while this study exclusively focused on BCG, future research should evaluate the feasibility of using SCG during exercise to estimate V̇O_2_ max. Previous studies have demonstrated the utility of SCG at rest in assessing cardiorespiratory fitness (20, 22). SCG-derived parameters could be used as additional inputs to the equation used in this paper to better estimate VO_2_ max.

## 5 Conclusions

This randomized study demonstrates the potential of BCG-based methods to predict V̇O_2_ max using submaximal exercise protocols, offering a viable alternative to traditional exhaustive testing in healthy young subjects. A specific incremental protocol design, which includes short and frequent breaks to ensure quality BCG measurements, is proposed and validated. BCG-based estimation of V̇O_2_ max, using only an age-based estimation of maximal HR (HR_est_), shows high accuracy, achieving minimal bias, strong correlations, and good reliability. Compared to traditional methods requiring maximal exhaustion for maximal workload or maximal heart rate assessment, the proposed approach offers similar precision while significantly lowering the physical and logistical demands of testing. Further validation is needed in diverse populations to confirm broader applicability and usefulness.

## Data Availability

The raw data supporting the conclusions of this article will be made available by the authors, without undue reservation.
