# Supplementary material for: Estimating V̇O2 max in healthy subjects without maximal effort: a novel protocol using ballistocardiography
Source: Front Sports Act Living. 2026 Jan 15;7:1717782. doi: 10.3389/fspor.2025.1717782 (PMC12852396; doi:10.3389/fspor.2025.1717782)
Supplement: Supplementary file 1 [file Supplementaryfile1.docx]

Supplementary Material

# Appendix A: method to detect linearity break between kinetic energy metric and workload

The analysis begins by excluding baseline points, as they often represent non-representative or noise-dominated data. The initial set of data points is pre-processed to identify a potential starting point for the linear regression model. This is determined by examining the differences between consecutive points in the kinetic output series. If negative differences are observed, the regression starts from the first point with a positive trend, ensuring the exclusion of points that might disrupt the linearity assumption.

An initial regression model is then fitted using three consecutive points, starting from the determined point of interest. A linear regression analysis is performed using these points, and the residuals from the model are calculated. The mean squared error (MSE) is computed to evaluate the goodness of fit. Subsequently, additional points are added iteratively to the model. At each step, a new regression model is fitted, and the SSR is recalculated to monitor the progression of fit quality.

The method detects the point where the linearity assumption breaks by evaluating the ratio of the current MSE to the MSE from the previous step. A ratio greater than one indicates a potential deviation from linearity. Once such a deviation is detected, all subsequent points are excluded from the regression analysis. This ensures that only the data segment adhering to a linear relationship is retained for modeling.

# Appendix B: VO2 max estimation equations

The equations from the FRIEND, the WASSERMAN, the FRIEND-ergometry, the Military Fitness Test (MILFIT), and the STORER studies were applied to the raw data of the participants in the present study to compare the different prediction models with the one proposed in the present work. The equations are described hereafter.

FRIEND:

${VO}_{2max} (\frac{ml}{kg.min})=79.9-0.39*age \left[ y \right]-13.7*sex- 0.280*weight [kg]$ (B1)

Where sex is 0 if male, 1 if female.

WASSERMAN (men):

${VO}_{2max}(\frac{ml}{min})=(0.032*height \left[ cm \right]-0.024*age [y])+0.019*weight [kg]-3.17$ (B2)

WASSERMAN (women):

${VO}_{2max}(\frac{ml}{min})=(0.032*height \left[ cm \right]-0.024*age [y])+0.019*weight [kg]-0.49$ (B3)

FRIEND-ergometry:

${VO}_{2max}=1.74*\left( Pmax \left[ W \right]*6.12*weight \left[ kg \right] \right)+3.5$ (B4)

MILFIT:

${VO}_{2max}=12.35*\frac{P_{max} \left[ W \right]}{weight \left[ kg \right]}+3.5$ (B5)

STORER (women):

${VO}_{2max}=9.39*Pmax \left[ W \right]+7.7*weight \left[ kg \right]-5.88*age [y]+136.7$ (B6)

STORER (men):

${VO}_{2max}=10.51*Pmax \left[ W \right]+6.35*weight \left[ kg \right] -10.49*age \left[ y \right]+ 519.3$ (B7)

# Appendix C: Results for estimates of V̇O_2_ max normalized by body mass

| V̇O_2_ max estimation | Input | Bias [95% limits of agreement]  (ml/min/kg) | Pearson’s r  and p | Lin’s CCC [95% confidence interval] | CV  (%) | MAPE (%) | SEE (ml/min/kg) |
| --- | --- | --- | --- | --- | --- | --- | --- |
| FRIEND | Anthropometrics | -0.12  [-18.21; 17.96] | r = 0.79;  p < 0.001 | 0.79  [0.49; 0.92] | 11.64 | 13.06 | 9.58 |
| WASSERMAN | Anthropometrics | -9.45  [-25.38; 6.48] | r = 0.86;  p < 0.001 | 0.67  [0.39; 0.84] | 18.00 | 26.05 | 13.20 |
| FRIEND-ERGO | W_max_ | -6.65  [-18.41; 5.11] | r = 0.93;  p < 0.001 | 0.82  [0.62; 0.92] | 12.18 | 15.56 | 9.47 |
| MILFIT | W_max_ | 0.46  [-10.57; 11.49] | r = 0.94;  p < 0.001 | 0.93  [0.81; 0.97] | 7.24 | 9.32 | 5.86 |
| STORER | W_max_ | -2.28  [-14.16; 9.61] | r = 0.92;  p < 0.001 | 0.90  [0.74; 0.96] | 8.96 | 10.86 | 6.75 |
| BCG with protocol B1 | W_max_ | -2.69  [-15.89; 10.51] | r = 0.95;  p < 0.001 | 0.92  [0.80; 0.97] | 9.54 | 13.12 | 7.56 |
|  | HR_max_ | -2.73  [-16.02; 10.55] | r = 0.96;  p < 0.001 | 0.92  [0.82; 0.96] | 9.82 | 13.24 | 7.62 |
|  | HR_est_ | -1.96  [-17.35; 13.43] | r = 0.87;  p < 0.001 | 0.84  [0.61; 0.94] | 12.05 | 15.59 | 8.42 |
| BCG with protocol B2 | W_max_ | -3.31  [-33.10; 26.48] | r = 0.70;  p < 0.001 | 0.63  [0.26; 0.84] | 19.16 | 24.09 | 16.17 |
|  | HR_max_ | -5.87  [-28.95; 17.21] | r = 0.72;  p < 0.001 | 0.65  [0.27; 0.85] | 16.90 | 23.08 | 13.75 |
|  | HR_est_ | -5.05  [-32.72; 22.62] | r = 0.67;  p < 0.001 | 0.61  [0.21; 0.83] | 18.41 | 24.57 | 15.62 |

Table C1 Comparison of methods to estimate V̇O_2_ max normalized by body mass, based on bias, limits of agreement, Pearson’s correlation coefficient (r) and associated p-values, Lin’s Concordance Correlation Coefficient (CCC) with 95% confidence intervals, coefficient of variation (CV), and mean absolute percentage error (MAPE). The methods include standard predictive models (FRIENDS, WASSERMAN, FRIEND-ERGO, MILFIT, and STORER) and ballistocardiography (BCG)-based estimates using Protocols B1 and B2 under different inputs: maximal workload (W_max_​), measured maximal heart rate (HR_max_), and estimated maximal heart rate (HR_est_). Lower bias, narrower limits of agreement, and lower CV and MAPE values indicate higher precision and accuracy.
